# Influence of Sex on Cognition and Peripheral Neurovascular Function in Diabetic Mice

**DOI:** 10.3389/fnins.2018.00795

**Published:** 2018-10-31

**Authors:** Baoyan Fan, Xian Shuang Liu, Alexandra Szalad, Lei Wang, Ruilan Zhang, Michael Chopp, Zheng Gang Zhang

**Affiliations:** ^1^Department of Neurology, Henry Ford Health System, Detroit, MI, United States; ^2^Department of Physics, Oakland University, Rochester, MI, United States

**Keywords:** sex, cognition, peripheral neuropathy, neurovascular, diabetes

## Abstract

Cognition impairment and peripheral neuropathy (DPN) are two major complications of diabetes. The aim of the present study is to investigate the effect of sex differences on cognition and DPN in diabetic mice. Male and female BKS.Cg-*m*+/+*Leprdb/J* (db/db) and db/m mice were used. At ages of 20 and 30 weeks, all animals were subjected to learning, memory and neurological function tests. Regional blood flow in footpad and sciatic nerves were measured using laser Doppler flowmetry. Our data showed that male db/db mice aged 20 weeks and 30 weeks spent significantly more time to locate the hidden platform in the correct quadrant and spent significantly less time exploring the cage with a new stranger mouse compared to aged-matched female db/db mice. Electrophysiological recordings showed that male db mice aged 30 weeks had significantly reduced motor and sensory nerve conduction velocity compared with females. Hot plate and tactile allodynia tests revealed that males exhibited significantly higher thermal and mechanical latency than females. Male db mice aged 30 weeks displayed significantly reduced blood perfusion in sciatic nerve and footpad tissues compared with females. In addition, compared with male and female non-diabetic db/m mice, db/db mice exhibited increased time spent on locating the hidden platform, decreased time spent on exploring the novel odor bead and an unfamiliar mouse, as well as showed significantly lower levels of blood flow, lower velocity of MCV and SCV, higher thermal and mechanical latencies. Blood glucose levels and body weight were not significantly different between male and female diabetic animals (age 30 weeks), but male db mice showed a higher serum total cholesterol content. Together, our data suggest that males develop a greater extent of diabetes-induced cognition deficits and peripheral neurovascular dysfunction than females.

## Introduction

According to the International Diabetes Federation in 2015, diabetes mellitus has become a global burden with an estimated 415 million people suffering with diabetes mellitus worldwide (IDA, 2015). Complications of diabetes result in increased morbidity, disability, and mortality and are considered as a threat for the economies of all countries. Diabetes is associated with accelerated cognitive decline (Said, 2007; Ruis et al., 2009), particularly in older individuals. Diabetes-associated factors, e.g., chronic hyperglycemia, hypertension, insulin resistance, and lipid disorders are pertinent determinants of diabetes driven cognitive dysfunction (Said, 2007; Ruis et al., 2009). Cognition has already been impaired at the early stage of diabetes (Ruis et al., 2009). Peripheral neuropathy (DPN) is the most common neuropathy of diabetes (both type-1 & type-2 diabetes) in industrialized countries, and DPN has a variety of clinical manifestations. The vast majority of patients with clinical diabetic neuropathy have a distal symmetrical form of the disorder that progresses with sensory and autonomic manifestations predominating (Said, 2007).

Currently, the only effective treatment for diabetic complications including cognitive impairment and DPN is glucose control (Kawamura et al., 2012; Ojo and Brooke, 2015). However, choices and preferences of therapeutic strategies of diabetes as well as adherence to lifestyle and pharmacological interventions differ in both sexes. In addition, drug therapy may have sex-specific side effects for diabetes (Beery and Zucker, 2011). These data suggest a critical role of specific sex differences for treatment of diabetes and its complications. In addition to therapy option, mounting data show that sex differences have a great impact on progression, epidemiology, pathophysiology, and outcomes of diabetes and complications (Arnetz et al., 2014; Kautzky-Willer et al., 2016). Sex hormones influence specific cognitive abilities (Torres et al., 2006; Hausmann et al., 2009; Hirnstein et al., 2014). To our knowledge, there are no studies of whether males and females with diabetes differ in cognitive impairment.

For DPN, clinical and experimental studies show that sex and age play an important role in determining the occurrence and progression of neuropathy (Aaberg et al., 2008; Dinh and Veves, 2008; Franconi et al., 2012; Smith-Vikos and Slack, 2012; Won et al., 2012; Tata et al., 2013; Ohta et al., 2014; Stenberg and Dahlin, 2014). DPN is more frequently seen in males than in females (Aaberg et al., 2008; Dinh and Veves, 2008; Beery and Zucker, 2011; Franconi et al., 2012; Ohta et al., 2014; Stenberg and Dahlin, 2014). In retrospective studies, sex differences were observed in the onset of DPN, with males developing neuropathy earlier than females (Aaberg et al., 2008). Males developed DPN at 63 years, approximately 4 years earlier than did females at 67 years (Aaberg et al., 2008). Furthermore, there isan increased frequency for men to develop a foot ulceration compared to women (Kiziltan et al., 2007; Dinh and Veves, 2008). In contrast, neuropathic pain and negative sensory symptoms are more frequent in female than in male patients (Kiziltan et al., 2007). However, very little data on onset, pathogenesis, and progression of DPN with reference to sex is available in the literature. The present study investigated whether sex as a biological variable, affects pathogenetic mechanisms of cognitive deficit and development of DPN in diabetes.

## Materials and methods

### Animals

All experimental procedures were carried out in accordance with NIH Guide for the Care and Use of Laboratory Animals and approved by the institutional Animal Care and Use Committee (IACUC) of Henry Ford Hospital. Male and female BKS.Cg-*m*+/+*Lepr*^*db*^*/J* (db/db) mice (Jackson Laboratories, Bar Harbor, Maine) aged 20 and 30 weeks were used. Age-matched heterozygotes mice (db/m), a non-penetrant genotype, were used as the control animals.

### Glucose, glycosylated hemoglobin (HbA1C) tests

Plasma glucose, total cholesterol (TC) and triglyceride (TG) were measured using glucose, total cholesterol and triglyceride test strips, respectively, (Ascensia Contour; Bayer, Zurich, Switzerland) once a week, and HbA1c levels (Quickmedical, Issaquah, WA) were measured every 2 weeks.

### Learning and memory assays

Cognitive assessments were performed on mice aged at 20 and 30 weeks. To minimize animal stress, individual cognitive tests were performed on different days, in which mice were subjected to one test per day.

#### Morris water maze test:

The mouse was placed in a swimming pool with water of a comfortable temperature (22–25°C) (Vorhees and Williams, 2006). The swimming pool was subdivided into 4 equal quadrants formed by imaging lines. At the start of each trial, the mouse was placed at 1 of 4 fixed starting points, randomly facing toward a wall (designated North, South, East, and West) and allowed to swim for 90 s or until it finds the platform, which is transparent and invisible to the animals. If the animal found the platform by spatial navigation, it was allowed to remain on it for 10 s. If the animal fails to find the platform within 90 s, it was placed on the platform for 10 s. Throughout the test period, the platform was located in the northeast quadrant 2 cm below water in a randomly changing position, including locations against the wall, toward the middle of the pool, or off center, but always within the target quadrant. If the animal is unable to locate the platform within 90 s, the trial was terminated and a maximum score of 90 s is assigned. If the animal reaches the platform within 90 s, the percentage of time traveled within the northeast (correct) quadrant is calculated relative to the total amount of time spent swimming before reaching the platform and employed for statistical analysis. The latency to find the hidden escape platform was also recorded and analyzed. Probe trials were conducted after the last training trials. The platform was removed and the mouse was allowed to swim for 90 s. The moving path and the time spent in the correct quadrant were recorded.

#### Social recognition memory test:

The experimental procedure consisted of three consecutive parts (Richter et al., 2005; Spinetta et al., 2008): **(1) Habituation (5 min):** The test mouse was placed in the middle chamber, sliding doors were opened and the mouse was allowed to explore all three chambers. The two side chambers contained an empty cylinder. The empty cylinder presented a novel inanimate object without social value. General activity, possible preference for a certain part of the apparatus (middle or one of the side chambers) and exploration of the cylinder were measured. **(2) Sociability (10 min):** After the habituation period, the sliding doors were shut and the test mouse was enclosed in the middle chamber. An unfamiliar mouse (stranger) were put into one of the cylinders and placed in one of the side chambers. The location for stranger was alternated, either to the left or right chamber of the social test box. The cylinder in the other chamber was empty. Following placement of stranger, the doors were opened, and the test mouse has access to all three chambers. Increased time spent in the chamber and in the perimeter around the cylinder with the stranger indicates preference for the social stimulus compared to the empty cage. For additional analysis, the 10 min period was also subdivided into two blocks of 5 min. **(3) Social discrimination (5 min):** Again, the test mouse was confined to the middle chamber. Another unfamiliar mouse (new stranger) was placed in the cylinder that was empty during the sociability test. The “old” stranger will remain in position in its cylinder and chamber. The sliding doors were opened and the test mouse has access to the side chambers. It is expected that the test mouse will spend more time with the new stranger than the old stranger. Increased time spent with the new stranger is a measure of the discriminative ability of the test mouse, and is also indicative of intact working memory.

#### Novel object recognition test:

The test included three steps (Leger et al., 2013): **(1) Prehabituation procedures:** Animals were removed from group-housing cages and rehoused singly in identical cages with sawdust bedding and removable wire tops. Once singly housed, animals remained in these test cages for the duration of the experiment. During the initial 24 h familiarization period, four 20 mm round wooden beads, each with a small hole bored through its diameter were introduced into the test cages without touching the beads in order to acquire the odor of the animal and to serve as familiar odors for subsequent use in the experiment. Housing the animals in the test cages with the beads for 24 h allowed for familiarization to both the testing environment and the presence of the beads. Several beads were also introduced into the cages of three selected odor-donor groups (housed three mice per cage), whose cages were not changed for 1 week to allow for a build-up of animal-specific novel odors. Wood beads incubated in these odor-donor cages provided equally novel odors for the following test. The cages designated to provide donor odor beads were counterbalanced, so that any one odor served as either a recently novel odor (N1) or a brand new novel odor (N2) during memory assessment for different experimental mice. **(2) Habituation to the first novel odor:** After 24 h of familiarization to the presence of four beads in the testing environment, the four now-familiar beads were removed 1 h before testing the mouse and stored in ziplock bags labeled and sealed as “F” (familiar) bead. Gloves were changed for every cage when removing beads from cages. After this 1-h period, a novel-odor wood bead (N1), taken from an odor-donor cage, and three familiar beads that were taken from their own cages 1 h prior were introduced into the cage (gloves were changed before handling beads from different cages). Mice were exposed to these four beads for three 1-min trial rounds with 1-min intervals during which the beads were removed from the testing enclosure. For each 1-min trial, three familiar-odor beads and the N1 bead were re-placed in different positions to avoid location effects (**Figure 2F**). The first approach to a bead made during this period initiated the timing of the 1-min trial. During the trials, exploration time for each of the four beads, defined as sniffing, licking, chewing, or having moving vibrissae while directing the nose toward and ≤1 cm from the object, was recorded separately using timers. N1 beads were discarded after habituation, and familiar beads were put back into the cage with the mouse. **(3) Odor-recognition memory assessment:** Twenty-four hours after the novel-odor habituation phase, the odor-recognition test was conducted on the mice aged 20 and 30 weeks. For this phase of the task, four familiar beads were removed 1 h before the test and stored in ziplock bags. One hour later, an odor N1 bead taken from the same cage as in habituation test and one unfamiliar novel-odor bead (N2) taken from a different odor-donor cage and two familiar (F, own-cage) odor beads were added to the mice (**Figure 2G**) following the same procedure outlined for the habituation phase. Only one round of the 1 min trial from when the animal starts sniffing any bead was conducted. All 4 beads were discarded after the test. The N2 ratio was calculated as time spent exploring the novel wood bead (N2) relative to the total time spent exploring all objects. The discrimination index (DI) was calculated as time spent exploring the novel objects compared with the familiar objects relative to the total time spent exploring all objects, according to the formula: (t[novel]-t[familiar])/(t[novel]+t[familiar])^*^100 (Stuart et al., 2013).

### Measurement of thermal sensitivity

To examine the sensitivity to noxious heat, plantar and tail flick tests were measured on mice aged 20 and 30 weeks using a thermal stimulation meter (IITC model 336 TG; IITC Life Science, Woodland Hills, CA) (Liu et al., 2017). For the plantar test, the meter was activated after placing the stimulator directly beneath the plantar surface of the hind paw. The paw-withdrawal latency in response to the radiant heat (15% intensity, cut-off time 30 s) was recorded. For tail-flick test, the meter was set at 40% heating intensity with a cut-off at 10 s. For both tests, at least five readings per animal were taken at 15 min intervals, and the average was calculated.

### Tactile allodynia test

Von Frey filaments were employed to stimulate paw withdrawal on mice aged 20 and 30 weeks (Liu et al., 2017). Briefly, a series of filaments were applied to the plantar surface of the left hind paw with a pressure causing the filament to buckle. A paw withdrawal in response to each stimulus was recorded and a 50% paw withdrawal threshold was calculated according to a published formula.

### Electrophysiology measurements

Sciatic nerve conduction velocity was assessed with orthodromic recording techniques on mice aged at 20 and 30 weeks, as previously described (Liu et al., 2017). During the measurements, animal rectal temperature was kept at 37°C using a feedback controlled water bath. Motor nerve conduction velocity (MCV) was calculated by dividing the distance between stimulating electrodes by the average latency difference between the peaks of the compound muscle action potentials evoked from 2 sites (sciatic notch and ankle). Sensory nerve conduction velocity (SCV) was calculated by dividing the distance between stimulating and recording electrodes by the latency of the signal from the stimulation artifact to the onset of the peak signal.

### Measurement of regional blood flow and plasma-perfused blood vessels

Regional sciatic nerve blood flow was measured in mice aged 30 weeks before the animals were sacrificed using a laser Doppler flowmetry (LDF PeriFlux PF4, Perimed AB, Sweden), as previously described (Liu et al., 2017). Relative flow values expressed as perfusion units (PU) were recorded under anesthesia. Regional sciatic nerve blood flow values from db/m mice were used as baseline values and data are presented as a perfusion ratio.

To further examine blood perfusion in foot pads and sciatic nerves, a laser Doppler perfusion imager system was employed and data were analyzed with PIMSoft Software (Perimed AB). Mice were anesthetized and the sensor was placed 10 cm above foot pad or exposed sciatic nerve. The apparatus displayed blood perfusion signal as a color-coded image ranging from dark blue (low perfusion) to bright red (high perfusion). To analyze the regional blood flow, we calculated time periods of interest and the average regions of interest (ROI) for each animal.

### Immunohistochemistry and image analysis

The sciatic nerve and epidermal foot pad tissues isolated from the animals aged 30 weeks were used for immunohistochemistry. Semi-thin sections (2 μm) stained with toluidine blue were used to analyze myelin thickness (Di Scipio et al., 2008). Morphometric analyses were performed using a MCID imaging system (Imaging Research Inc, St. Catharines, ON, Canada) according to our published protocols (Liu et al., 2017).

To measure intraepidermal nerve fibers (IENFs), the antibody against protein gene product 9.5 (PGP9.5, 1:1,000; Millipore) was applied on foot pad tissue sections. IENF profiles were imaged under a 40 × objective (Carl Zeiss, Germany) via the MCID system. The number of nerve fibers crossing the dermal-epidermal junction was counted and the density of nerves was expressed as fibers/mm length of section. Representative images of intraepidermal nerve fibers were obtained using a laser-scanning confocal microscope (LSCM, Olypus FV2000, Olympus Corporation, Japan).

### Statistical analysis

The data are presented as mean ± SE. Two-way analysis of variance followed by the Student-Newman-Keuls test were performed for multiple sample analysis. The analysis of covariance with repeated measure was used to test the effect of sex difference on functional recovery by considering possible interactions between groups (db/db) and various time points. A value of *P* < 0.05 was taken as significant.

## Results

### Metabolic parameters in male and female animals

To test the effects of sex on cognition and neurovascular function in diabetic mice, male and female diabetic (BKS.Cg-*m*+/+*Leprdb/J*, db/db) and non-diabetic (db/m) mice aged 20 and 30 weeks (*n* = 10/group/age) were employed. We first evaluated the metabolic properties in the circulation. There was no statistical difference in blood glucose, HbA1C levels and body weight between male and female diabetic animals (age 30 weeks), but male db/db mice showed a higher serum total cholesterol content (Table 1, *P* < 0.0001). In line with previous publication, blood glucose, HbA1C levels and cholesterol content were significantly higher (*P* < 0.0001) in diabetic db/db mice than those in control non-diabetic db/m mice.

**Table 1:** Concentrations of blood glucose and lipids in diabetic and non-diabetic mice.

	**dm male**	**dm female**	**db male**	**db female**
Glc(mg/dl)	110 ± 8.5	104 ± 10.3	459 ± 24.5^#^	487 ± 31.2
HbA1c(%)	4.0 ± 0.2	4.1 ± 0.3	10.3 ± 0.5^#^	10.1 ± 0.4
TC(mg/dl)	100.2 ± 9.8	93.5 ± 10.6	160.1 ± 11.5^#^	148.5 ± 12.8^*^
TG(mg/dl)	50.1 ± 7.9	52.2 ± 6.7	79.7 ± 10.3^#^	75.2 ± 6.2

* *P < 0.05, db male group vs. db female group, N = 10/group*.

# *P < 0.05, dm male vs. db male*.

### Male diabetic mice demonstrate more severe learning and memory deficits than female mice

**Figure 1:**
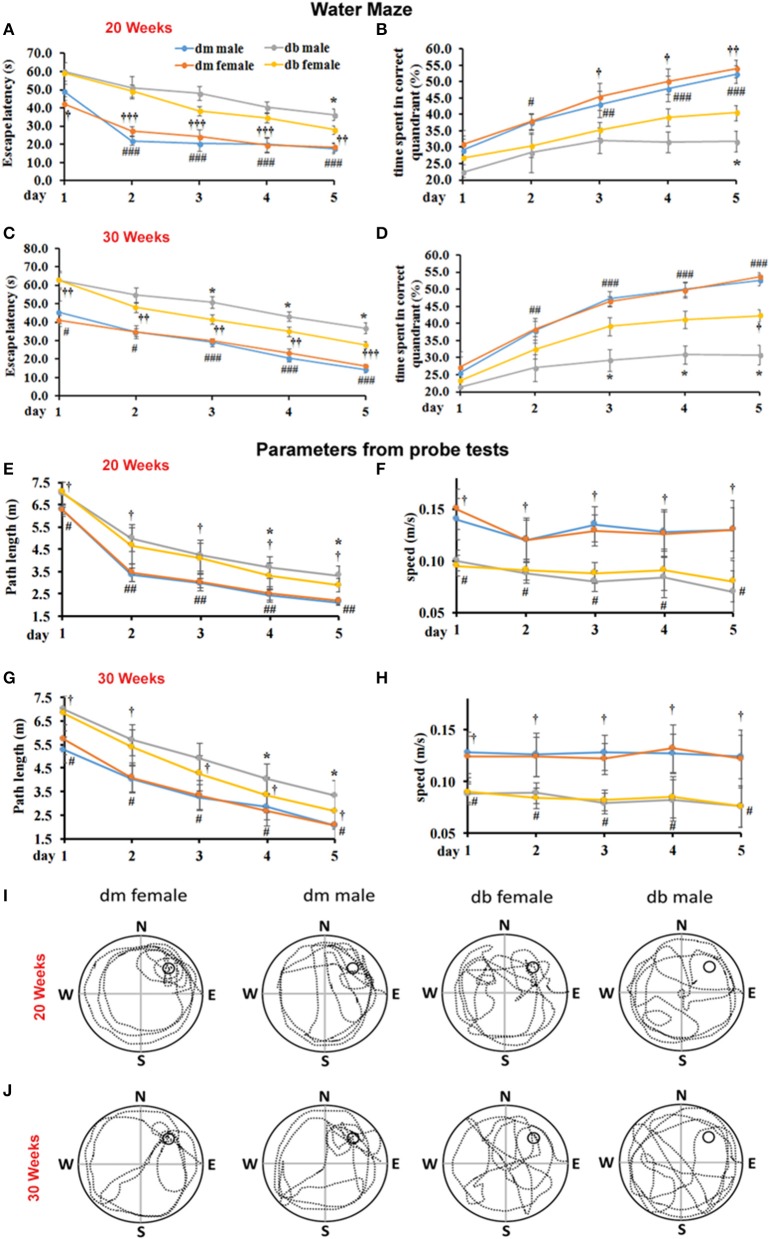
The effect of sex difference on spatial memory performance. Morris water maze was used to test the spatial learning and memory. Male db/db mice aged 20 **(A,B)** and 30 weeks **(C,D)** spent significant time to locate the hidden platform in the correct quadrant as indicated by quantitative latency compared to female diabetic mice **(A,C)**. Male db/db mice also spent much less time in the correct quadrant **(B,D)**. Also, db/db mice aged 20 **(A,B)** and 30 weeks **(C,D)** spent significant time to locate the hidden platform in the correct quadrant and much less time in the correct quadrant compared with aged-matched non-diabetic db/m mice. Panel bar graphs show path length (cm, **E,G)** and swimming speed (m/s, **F,H)** in animals aged 20 **(E,F)** and 30 weeks **(G,H)**. Representative swimming paths of mouse trajectories on the probe trial in the female or male diabetic db/db and non-diabetic db/m mice aged 20 **(I)** and 30 weeks **(J)**. *P < 0.05; ^#^*P* < 0.05, ^##^*P* < 0.01 dm male vs. db/db male, ^###^*P* < 0.001 dm male vs. db/db male; *P* < 0.05 dm female vs. db/db female, *P* < 0.001, *P* < 0.001 dm female vs. db/db female. *N* = 10 per group.

Cognitive dysfunction with its wide range, from mild cognitive impairment (MCI) through dementia, is one of the chronic complications of diabetes (2, 3). The effect of sex on cognitive function in diabetic animals is unknown. Using a Morris water maze assay that detected spatial learning and memory, we found that male db/db mice aged 20 weeks (Figures 1A,B) and 30 weeks (Figures 1C,D) spent significantly more escape latency time to find the hidden platform (*P* < 0.05) than aged-matched female db/db mice. In addition, male db/db mice spent significantly less time in target correct quadrant (%) than female db/db mice (Figures 1B,D). Compared with db/m non-diabetic mice aged 20 and 30 weeks, male and female diabetic db/db mice markedly increased escape latency time to locate hidden platform and decreased the % time spent in the target quadrant where the platform located (Figure 1). During the probe trial, male db/db mice showed more path length in the pool quadrant than female db/db mice aged 20 (Figures 1E,I) and 30 weeks (Figures 1G,J). These results of the probe trial suggest that spatial memory is impaired in male db/db mice compared with female db/db mice. No significant difference was observed in swimming speed of mice between the two groups, implying that swimming ability did not bias the results of training tasks or the probe trial (Figures 1F,H).

**Figure 2:**
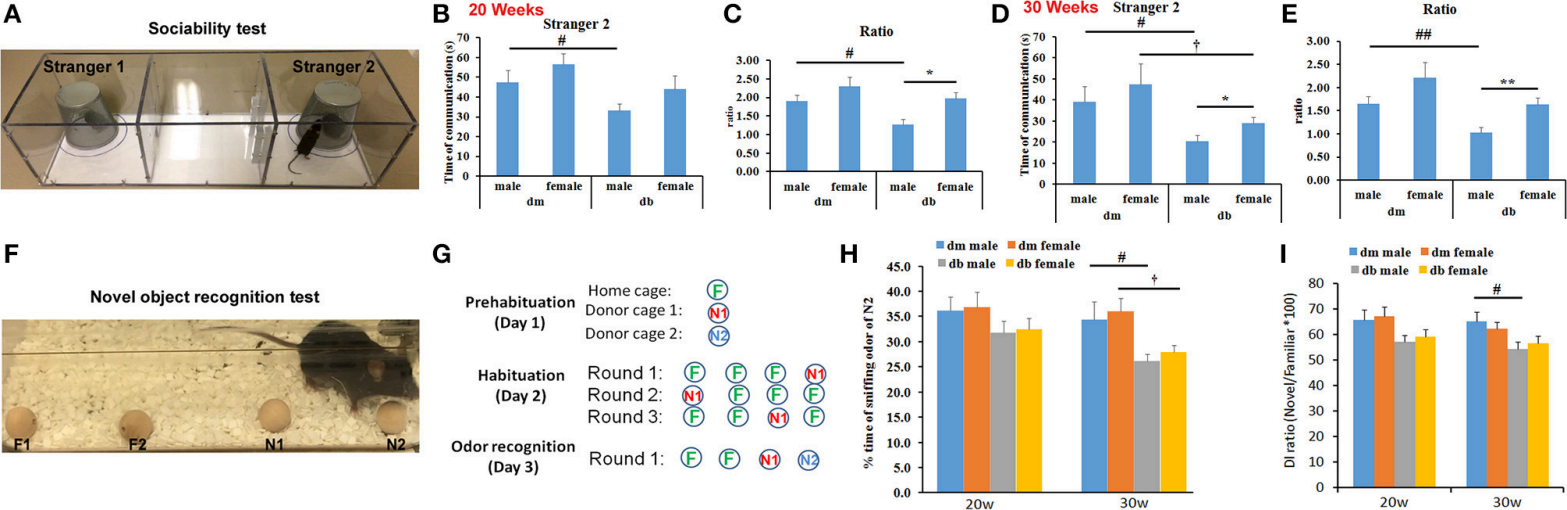
Social discrimination and recognition memory between males and females. A three-chambered box **(A)** and wooden beads **(F)** were used for social novelty test **(A–D)** and odor–based novelty recognition test **(F–I)**, respectively. Male db/db mice spent significantly more time on exploring a new stranger mouse (**B–E**, stranger 2), compared to female db/db mice. But there is no significant difference in time spent on a novel bead (**H**, N2 bead) and novel object discrimination index (DI, **I**) between male and female db/db mice. Compared with male and female non-diabetic db/m mice, db/db mice decreased time spent on exploring an unfamiliar mouse and recognizing the novel odor bead **(B–E)**. The ratio **(C,E)** refers to the time spent on a new stranger mouse (stranger 2) divided the time spent on an old stranger mouse (stranger 1). Illustration of the experimental approach (circles correspond to odor beads and familiar, novel beads were labeled as green “F” and red or blue “N”, respectively). Data in novelty recognition test **(H)** were presented as the percentage of time spent on N2 relative to the total amount of time spent with all beads. N1 = familiar odor bead; N2 = unfamiliar novel odor bead. *P < 0.05, **P < 0.01 db/db male vs. db/db female; ^#^*P* < 0.05, ^##^*P* < 0.01 dm male vs. db/db male; *P* < 0.05 dm female vs. db/db female. *N* = 10 per group.

The social odor–based recognition task was employed to detect sociability and non-spatial memory deficits. Using the three-chambered social approach test, this test is sensitive and permits measurement of sociability and preference for social novelty (Figure 2A). We found that male and female db/db mice aged 20 and 30 weeks showed different preferences (Figures 2B–E). The male db/db mice spent significantly less time exploring the cage in which a new stranger mouse existed (stranger 2) than female db/db mice, but the male and female db/m mice spent a comparable amount of time in the chamber with the new stranger mouse (Figures 2A–E). In addition, compared to non-diabetic db/m mice, diabetic male and female db/db mice aged 30 weeks spent significantly less time around the cage with a new stranger mouse than with a familiar mouse (Figures 2A–E). These results suggest a lack of social interaction with a novel mouse over a familiar mouse in both male and female diabetic mice compared with non-diabetic mice, and lack of sociability being more pronounced in male vs. female db/db.

Using a novel object recognition assay (Figures 2F–I), we did not find a significant difference in the time spent exploring a novel bead and discrimination index between male and female diabetic mice aged 20 and 30 weeks, respectively (Figures 2H,I, Supplementary Videos 1, 2). However, compared to db/m non-diabetic mice, diabetic male and female db/db mice exhibited reduced responses to a new bead (Figure 2H), suggesting that diabetes significantly impairs the animals' ability to discriminate the novel from the familiar object.

### Male diabetic mice exhibit worse neurological deficits

**Figure 3:**
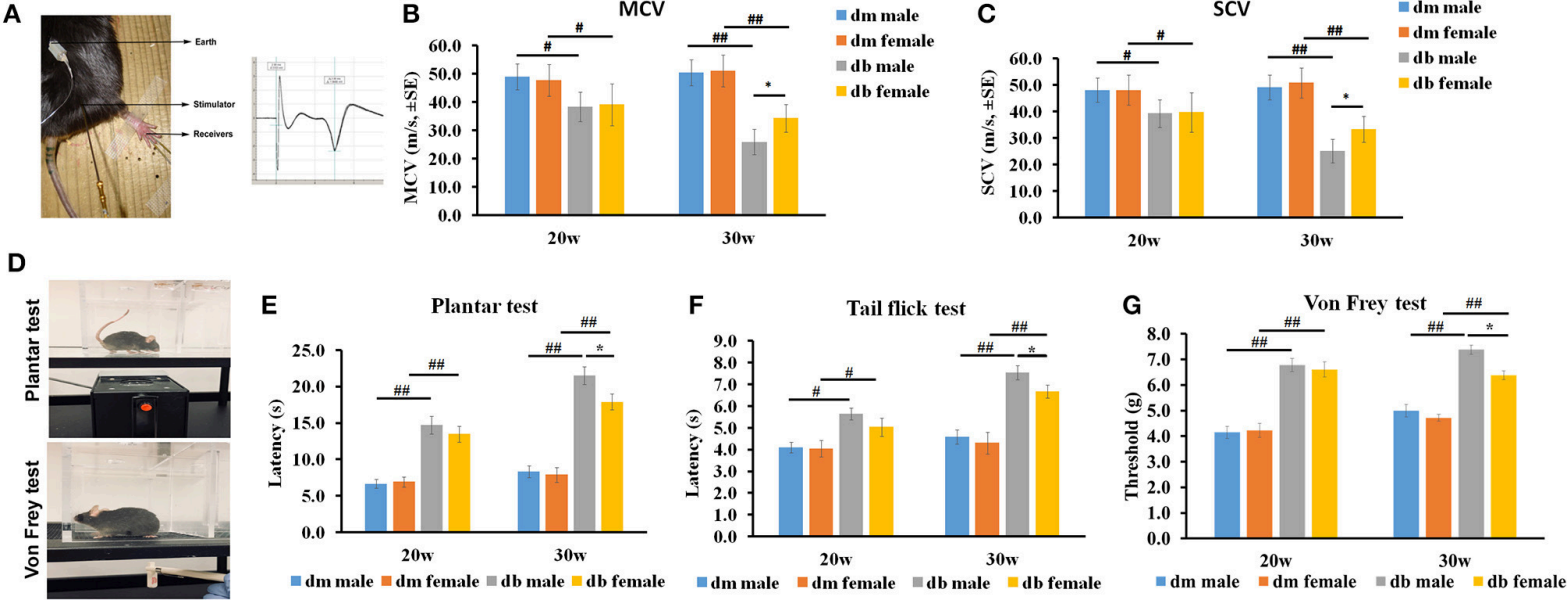
The differences of nerve conduction velocity and behavioral assays between males and females. **(A)** shows the electrophysiological recording of stimulated sciatic nerve in mice aged 20~30 weeks. Analysis of motor (**B**; MCV) and sensory (**C**; SCV) nerve conduction velocity shows that male diabetic mice displayed lower MCV and SCV velocity than female db/db mice at 30 weeks but not at 20 weeks, indicating that male diabetic mice have severe electrophysiological deficits. Both male and female db/db mice demonstrated decreased MCV and SCV compared to age matched male and female db/m mice. **(D)** Shows the IITC Plantar Analgesia Meter and Von Frey test system. **(E-G)** show that male diabetic mice exhibited increased thermal latency and thresholds for mechanical stimuli than female diabetic mice aged 30 weeks as measured by Plantar test **(E)**, Tail flick test **(F)**, and Von Frey test **(G)**. Both male and female db/db mice showed increased thermal latency, decreased mechanical sensitivity compared to age matched male and female db/m mice. ^#^*P* < 0.05, ^##^*P* < 0.01 db/m male group vs. db/db male group, db/m female group vs. db/db female group; *P < 0.05, db/db male group vs. db/db female group, *N* = 10/group. dm = db/m mouse; db = db/db mouse.

We then evaluated if sex affected the neurological function of diabetic mice aged 20 and 30 weeks in an age-dependent manner. Motor (MCV) and sensory (SCV) nerve conduction velocities were measured using electrophysiological recordings (Figure 3A). Electrophysiological recordings showed that male db/db mice at age of 30 weeks had significantly reduced motor (Figure 3B, 26.0 ± 4.4 mm/s) and sensory nerve conduction velocities (Figure 3C, 25.1 ± 5.8 mm/s), compared with female db/db mice (MCV: 34.3 ± 4.9 mm/s, *P* < 0.05; SCV: 33.3 ± 4.9 mm/s, *P* < 0.05, Figures 3B,C). At 20 weeks, all db/db mice had developed DPN, but no significant differences of MCV and SCV were observed between male and female diabetic mice. However, compared to aged matched db/m mice, db/db mice at age 20 and 30 weeks exhibited a significant reduction of MCV and SCV (Figures 3B,C).

Next, we examined thermal and mechanical function in male and female diabetic mice. Results of hot plate, tactile allodynia and von Frey tests (Figure 3D) revealed that, male db/db mice exhibited a significantly higher thermal (male vs. female: 21.5 ± 1.2s vs. 17.9 ± 1.1s, *P* < 0.05, Figure 3E) and mechanical latencies (7.4 ± 0.8s vs. 6.3 ± 0.7s, *P* < 0.05, Figure 3F) than female db/db mice aged 30 weeks. There was a slightly higher thermal and mechanical latency in male db/db mice than for females at 20 weeks (Figures 3E–G). Consistent with a prior publication (Liu et al., 2017), thermal and mechanical latency was significantly elevated in db/db mice at age 20 and 30 weeks compared to aged matched db/m mice (Figures 3E–G).

### Sex differences of blood perfusion in diabetic mice

**Figure 4:**
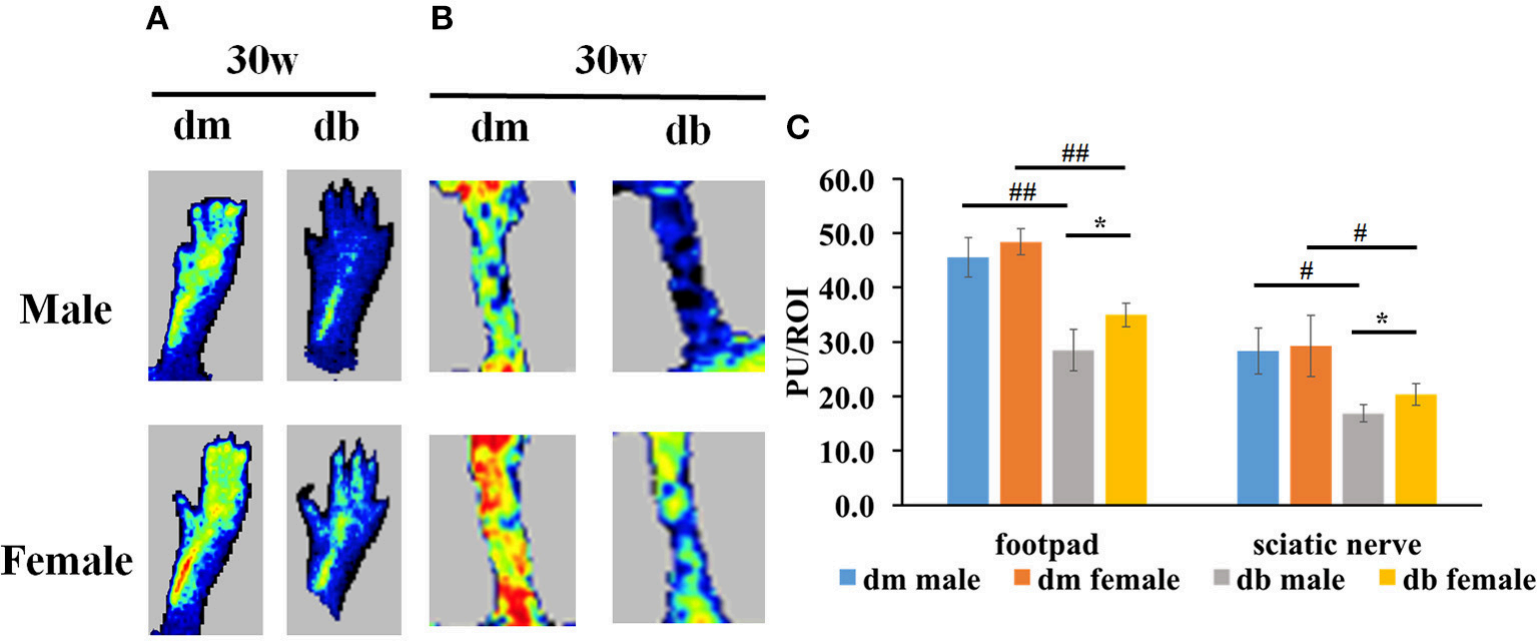
The differences of regional blood flow between males and females. Laser Doppler images show that male diabetic mice displayed a significant reduction of regional blood flow in footpad **(A)** and sciatic nerve **(B)** tissues compared to female diabetic mice. **(A,B)** show representative images of regional blood flow in foot pad and sciatic nerve tissues. **(C)** shows the quantitative data of regional blood flow. PU, Perfusion units; ROI, Region of interest. ^#^*P* < 0.05, ^##^*P* < 0.01 db/m male group vs. db/db male group, db/m female group vs. db/db female group; *P < 0.05, db/db male group vs. db/db female group. *N* = 10/group. Lowest blood flow is indicated in blue, maximum blood flow in red, and intermediate grading in green and yellow.

Vascular dysfunction precedes the appearance of nerve conduction velocity deficits (Coppey et al., 2001, 2002). Damaged microvasculature supplying the peripheral nerves leads to impairment of the nerve fibers and eventually to symptoms of DPN. To examine if sex difference impacted on the regional blood flow in diabetic animals, laser Doppler flowmetry (LDF) was used. We found that db/db mice at age of 30 weeks showed a significant reduction of blood flow in foot pad (Figure 4A) and sciatic nerve tissues (Figure 4B) measured with LDF in male compared to female db/db mice (Figure 4). Compared to aged matched db/m mice, db/db mice exhibited a significant reduction of blood flow (Figure 4).

### Sex differences in nerve function and axonal myelination

**Figure 5:**
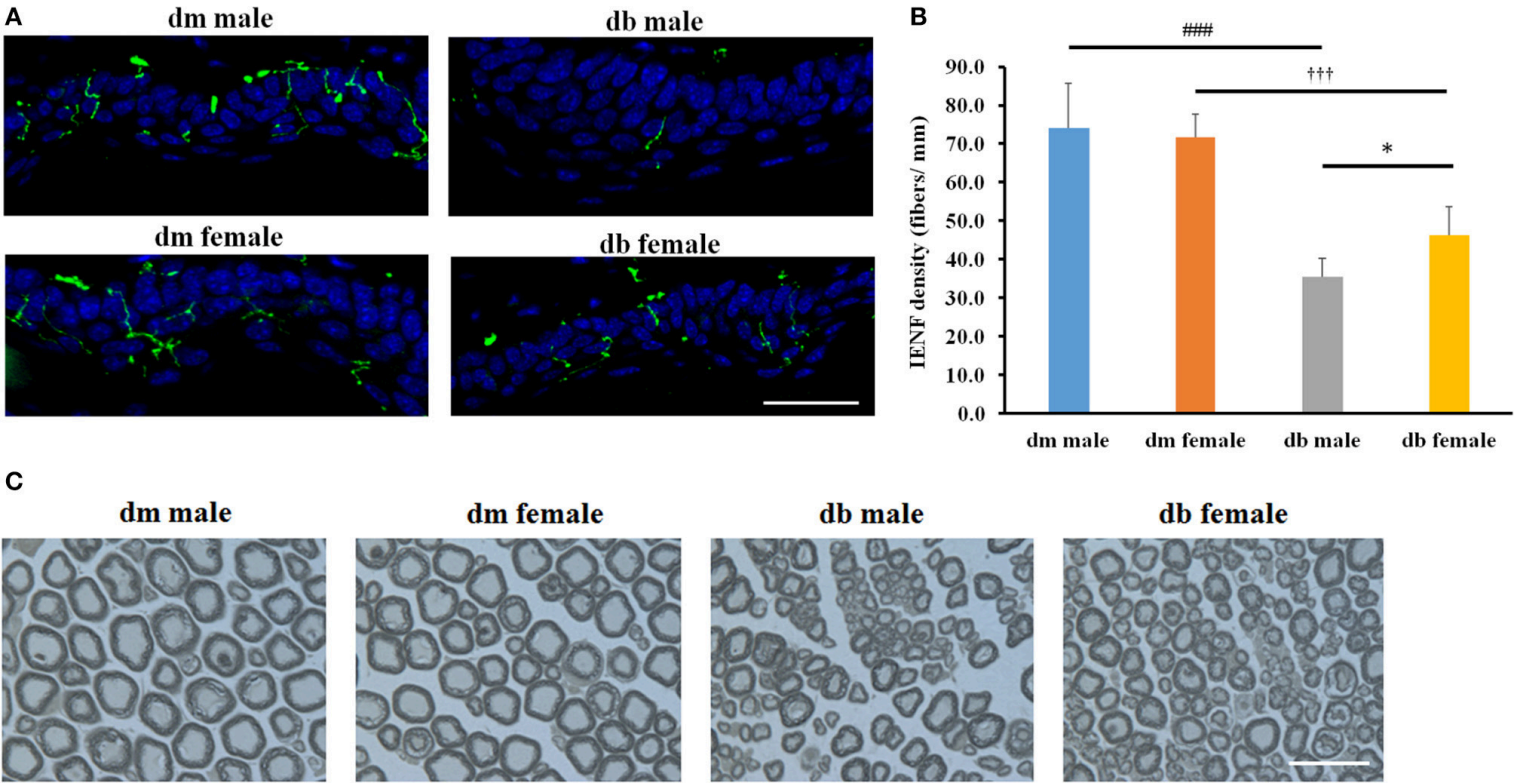
The differences of epidermal innervation and morphometric changes of myelinated sciatic nerves between males and females IENF. Representative immuno-fluorescent images show PGP9.5 staining IENF (green, arrows) and epidermal innervation in the hind plantar paw skin of in mice aged 20~30 weeks **(A)**. Histogram represents the mean number of IENF per site **(B)**. Representative images of semi-thin toluidine blue-stained transverse sections of sciatic nerves derived from male and female db/db or db/m mice aged 20–30 weeks **(C)**. *P < 0.05, db/db male vs. db/db female; ^###^*P* < 0.001 db/m male vs. db/db male; *P* < 0.001 db/m female vs. db/db female. Bar = 50 μm. *N* = 10/group.

Intraepidermal nerve fibers (IENF) innervate dermis and epidermis, and a measurement of IENF density through skin biopsy has been widely used in the clinical diagnosis of peripheral neuropathy and in monitoring its response to treatment (Tesfaye et al., 2010). We analyzed the IENF densities of the foot pads. Morphological analysis was performed to assess the effect of sex on IENF innervation and quantification of IENF densities in diabetic mice. Significant differences were found between male and female db/db mice with significantly increased number of PGP9.5 positive IENFs in male db/db mice compared to female mice (*P* < 0.05; Figures 5A,B). There were also distinctions between control db/m and diabetic db/db mice (*P* < 0.001), where the number of PGP9.5 positive IENFs was significantly decreased in male and female diabetic mice compared to db/m sex-matched mice, respectively, while no difference was observed between male and female db/m non-diabetic mice (Figures 5A,B).

Microvascular dysfunction accompanies demyelination and severe loss of myelinated axons in peripheral nerves, which are related to the progression of DPN. To examine whether microvascular injury difference accompanies the demyelination and axonal loss due to the sex difference, we measured myelinated sciatic nerves on semi-thin transverse sections stained with toluidine blue. Myelinated fiber diameter and myelin sheath thickness were measured using a MCID image analysis software, and g-ratio was calculated to determine the degree of myelination. We found significant reductions of myelin thickness, axon and fiber diameters and induction of g ratio in male compared with female db/db mice aged 30 weeks (Figure 5C, Table 2). Consistent with previous studies, decreased myelin thickness, axon and fiber diameters and increased g ratio were observed in db/db mice compared with db/m mice in both male and females (Figure 5C, Table 2); however, axonal myelination was not significantly different between male or female non-diabetic db/m mice.

**Table 2:** The quantitative data of histomorphometric parameters of sciatic nerves.

**Property**	**dm male**	**dm female**	**db male**	**db female**
Fiber diameter(μm)	9.10 ± 0.32^###^	8.96 ± 0.27^^†††^^	8.10 ± 0.40	7.24 ± 0.29^**^
Axon diameter(μm)	5.24 ± 0.22^^#^^	5.16 ± 0.22^^†††^^	4.87 ± 0.22	4.56 ± 0.21^*^
myelin thickness(μm)	1.91 ± 0.10^^#^^	1.90 ± 0.09^^†††^^	1.61 ± 0.13	1.35 ± 0.06^**^
g ratio	0.58 ± 0.01^###^	0.58 ± 0.02^^†^^	0.60 ± 0.02	0.63 ± 0.01^*^

* *p < 0.05*,

** *p < 0.01, db male vs. db female*.

# *p < 0.05*,

### *p < 0.001 dm male vs. db male*.

† *p < 0.05*,

††† *p < 0.001 dm female vs. db female*.

*Bar = 25 μm, N = 10/group*.

## Discussion

Few studies have highlighted sex differences in diabetic cognitive impairment and neuropathy. In the present study, we examined whether cognitive deficits and neurovascular function differed in female and male diabetic mice. Compared with female diabetic db/db mice, male diabetic db/db mice exhibited more severe learning and memory impairments than female diabetic mice aged 30 weeks. Furthermore, male db/db mice showed significantly lower levels of blood flow, lower MCV and SCV as well as higher thermal and mechanical latencies than female db/db mice. Our data suggest that males develop more pronounced age-dependent cognitive and neurovascular dysfunction in DPN.

Diabetes is associated with accelerated cognitive decline and an increased risk of dementia (Said, 2007; Ruis et al., 2009). To our knowledge, it remains unclear if diabetes-induced cognition impairment is sex-specific. Our diabetic db/db model aged 20 and 30 weeks showed significant learning and memory impairment compared with non-diabetic db/m mice. Our results further demonstrated that male diabetic mice aged 20 and 30 weeks spent more time in the target quadrant of the Morris water maze and less time with a new stranger mouse in social interaction cognitive tasks, compared with age-matched female diabetic mice, indicating increased social and cognitive dysfunction in the male diabetic mouse. The biological and cerebral structural bases underlying cognition difference between male and females remain elusive. Hippocampal integrity is critical for spatial memory (Broadbent et al., 2004). Generation of newborn neurons in the hippocampus is involved in spatial learning and memory (Clelland et al., 2009; Aimone et al., 2011). The medial nucleus of the amygdala is one of the best documented regions involved in social interactions (Hong et al., 2014; Barak and Feng, 2016). Moreover, the hippocampus and the perirhinal cortex play an important role in the process of object recognition and memory (Antunes and Biala, 2012). It was previously reported that sex difference can affect learning, synaptic activity, and long-term potentiation in the hippocampus (Maren et al., 1994; Monfort et al., 2015; Qi et al., 2016). Further investigation of these brain structures and their functions may help to understand the difference of diabetes-induced cognition impairment in a sex-dependent manner.

Db/db mice aged 20 weeks were chosen as the starting point for evaluation of sex differences since onset of peripheral neuropathy occurs at this time point (O'Brien et al., 2014; Yorek et al., 2015). At age of 30 weeks, we observed a more severe mechanical hypoalgesia, thermal allodynia and thermal hyperalgesia in male diabetic db/db mice compared with females. Brien et al reported that similar motor and sensory NCV deficits were observed in male and female db/db mice aged 24 weeks (O'Brien et al., 2016). We demonstrated the severe reductions in MCV and SCV in male diabetic db/db mice compared with female mice aged 30 weeks, but not 20 weeks. These data suggest that both sex and age play important roles in determining the occurrence and progression of neuropathy, which is relevant to clinical and experimental studies showing that DPN is more frequently seen in males and also there is an increased frequency for men to develop a foot ulceration compared to women (Dinh and Veves, 2008).

The development and progression of DPN are closely associated with marked neurovascular abnormalities (Coppey et al., 2001, 2002). We thereby examined the blood perfusion with LDF. Our data demonstrated that male db/db mice aged 30 weeks displayed significantly reduced blood flow in the sciatic nerve and foot pad than female db/db mice, suggesting that diabetes leads to damage of vessels that supply nerves differently in males and in females. A decrease of blood vessel growth has been shown to be connected with axonal regeneration in skin biopsy of DPN patients (Lauria and Lombardi, 2007). We therefore examined the effect of sex on IENF in diabetic mice. Our data clearly showed a greater IENF density in female db/db mice than their male counterparts in female diabetic mice with DPN. Also, we found the decreased myelin thickness, axon and fiber diameters and increased g ratio were more prominent in male db/db mice compared with female db/db mice. These data demonstrate an increased axonal demyelination in male compared with female diabetic mice. The extent of neuronal damage resulting from diabetes was sex-specific with female animals being less sensitive than males, suggesting that the male peripheral nerves exhibit a more hyperglycemia-sensitive phenotype than the female peripheral neurons. Our previous study demonstrated that myelin thickness and fiber diameter in male db/db mice aged 20 weeks were further reduced at age of 28 weeks compared with age-matched male db/m mice (Liu et al., 2017). A caveat in our study is that all animals were sacrificed at the age of 30 weeks, so we did not compare myelination between males and females at 20 and 30 weeks. Further studies comparing the differences in myelination between male and female diabetic mice at 20 and 30 w are warranted.

The molecular mechanisms why male subjects with diabetes develop cognitive deficit and neuropathy earlier than female subjects are unclear and may be complex, but factors such as testosterone deficiency, which is common in men with diabetes, may contribute to the discrepancy in development, at least in type 2 diabetes in humans (Kamenov et al., 2010). In line with human study, it has been recently shown that neuroactive steroid levels in male diabetic animals cause an alteration of their mitochondrial function that subsequently affects axonal transport, contributing to the sex difference in DPN (Pesaresi et al., 2018). Interestingly, neuroactive steroid treatment such as progesterone and testosterone on DPN suggests that the efficacy differs between sex which is related to neuroactive steroid levels (Roglio et al., 2008; Melcangi et al., 2011; Giatti et al., 2015). Elevated triglycerides are related to the decreases of IENF and contribute to DPN progression in diabetic patients (Vincent et al., 2009a,b; O'Brien et al., 2016). Our data revealed a significant difference in total cholesterol (TC) levels in male and female db/db animals, suggesting that dyslipidemia may underline the sex dimorphisms in diabetes which may impact on DPN. In addition, differences between males and females in inflammation, oxidative reaction and myelin repair may also play important roles in cognitive deficit and DPN (Saltevo et al., 2009; Stenberg and Dahlin, 2014; Kautzky-Willer et al., 2016; Kander et al., 2017). Further research into the alteration of these factors in diabetic animals is warranted to unravel the underlying reasons for sex difference.

In conclusion, this study demonstrates that sex affects the development of cognitive impairment and peripheral neuropathy, with male diabetic mice having a greater extent of peripheral neurovascular dysfunction, loss of myelinated axons and intraepidermal nerve fibers than do female diabetic mice. Also, our study provides the first important evidence that sex difference plus diabetes not only affects the peripheral nerves but also the brain. Our data suggest that it is important to take into account sex differences as well as type of experimental diabetic model when new strategies and techniques for treatment of DPN are developed.

## Author contributions

All authors listed have made a substantial, direct and intellectual contribution to the work, and approved it for publication.

### Conflict of interest statement

The authors declare that the research was conducted in the absence of any commercial or financial relationships that could be construed as a potential conflict of interest.

## Supplementary material

Supplementary Videos 1, 2 Novel object recognition assay showing that female diabetic db/db mice (Video) spent more time to explore a new novel wooden bead. However, compared to female mice, male mice (Video) significantly lost their focused responses to a new bead. The mice were presented with the odor N1 bead in the presence of one unfamiliar novel-odor bead (N2) taken from a different odor-donor cage and two familiar (own-cage) odor beads. The total measurement time is 1 min. Trials of exploration, defined as sniffing, licking, chewing, or having moving vibrissae while directing the nose toward and ≤1 cm from the wood bead, were recorded separately using stopwatches.
